# Tests of trend between disease outcomes and ordinal covariates discretized from underlying continuous variables: simulation studies and applications to NHANES 2007–2008

**DOI:** 10.1186/s12874-018-0630-7

**Published:** 2019-01-05

**Authors:** Naomi C. Brownstein, Jianwen Cai

**Affiliations:** 1 Department of Behavioral Sciences and Social Medicine, College of Medicine, Florida State University, Tallahassee, Florida, USA; 2 Department of Statistics, Florida State University, Tallahassee, Florida, USA; 3 Department of Biostatistics, Gillings School of Global Public Health, University of North Carolina at Chapel Hill, Chapel Hill, North Carolina USA

**Keywords:** Discretization, Linear regression, Power, Weighted least squares

## Abstract

**Background:**

Many epidemiological studies test trends when investigating the association between a risk factor and a disease outcome. Continuous exposures are commonly discretized when the outcome is nonlinearly related to exposure as well as to facilitate interpretation and reduce measurement error. Guidance is needed regarding statistically valid trend tests for epidemiological data of this nature.

**Methods:**

The association between a discretized variable and a disease is modeled through logistic regression or survival analysis. Linear regression is then conducted by regressing the odds ratio or relative risk on the midpoint of the exposure interval. The trend test is based on the slope of the regression line. In order to investigate the performance of this approach, we conducted simulation studies, considering ten different approaches for the linear regression based on the inclusion or exclusion of an intercept in the model and the form of the weights. The proposed methods are applied to the National Health and Nutrition Examination Survey (NHANES) 2007–2008 for illustration.

**Results:**

The simulation studies show that eight of these methods are valid, and the relative efficiency depends on the underlying relationship between the covariate and the outcome.

**Conclusions:**

The significance of the study is its potential to help practitioners select an appropriate method to test for trend in their future studies that utilize ordinal covariates.

## Background

Tests of trend are important in showing monotone relationships between risk factors and disease outcomes. Drug developers aim to demonstrate that efficacy and toxicity risk increase with dosage. In epidemiological studies, investigators may ask if disease prevalence increases with exposure. Examples include trends between observed vitamin D serostatus and change in adiposity indicators [1] and between physical activity level and decreasing breast cancer risk [2]. Investigating trends requires care; popular trend tests may not test for monotonicity [3]. Motivated by these applications, we investigate trend tests useful in epidemiology.

Logistic and Cox regression are often used for modeling the relationship between a disease outcome and a continuous risk factor. Yet, covariates may be subject to substantial measurement error for which it may be difficult to adjust [4, 5]. Coefficients for continuous predictors may be difficult to interpret in certain situations. Consequently, continuous variables are frequently discretized, for example, nutrient quantiles. In some settings, such as meta-analyses, it may be impossible to recover the underlying continuous values.

Tests of trend may be conducted based on rank statistics using only the ordinal group membership [6, 7]. Le et al. [8] propose a rank-based trend test between the ordinal covariates and the hazard function. Tarone [9] proposed an exact test for monotone trend in the hazard function, but this assumes identical censoring distributions for all groups. Although their lack of assumptions may render these tests seemingly appealing, they may be inappropriate and have low power when the ordinal covariate is non-uniformly distributed [10]. The widely-used Cochran-Armitage trend test may have inflated type I error when the covariate margins are unbalanced, even for large samples [11].

When a continuous exposure is divided into intervals and treated as ordinal, the median of each interval may serve as a regression model covariate. Medians of intervals defined by macronutrient quantiles were used to test for linear trends between dietary patterns and diabetes incidence [12]. Another common practice is to fit a regression model, treating the ordinal covariate as a categorical variable. Trend is assessed by examining whether the odds ratios or hazards ratios are changing monotonically with covariate level.

In this paper, we evaluate ten approaches to test for a trend in the odds ratios or hazard ratios. We present type I error and power through simulations and apply the methods to the NHANES dataset for illustration.

## Methods

We compare the type I error and power of trend tests based on weighted and unweighted regression. The underlying continuous covariate is exemplified using measurements of body mass index (BMI). We discretized observations into weight classes defined by the World Health Organization [13], as shown in Table 1.

**Table 1:** Description of NHANES Data by BMI Class

BMI Class	WHO Defined Lower Limit (kg/m^2^)	WHO Defined Upper Limit (kg/m^2^)	Number of Individuals	Unweighted Percent of Sample	WHO Defined Median (kg/m^2^)	Sample Median BMI (kg/m^2^)
Underweight	12	18.5	93	1.7	17	17.8
Normal Weight	18.5	25	1513	27.3	23	22.7
Overweight	25	30	1919	34.6	28	27.5
Obese	30	70	2026	36.5	35	34.0

Suppose that an ordinal covariate has *r* levels. Let the effect size, *θ*_*j*_, denote the odds ratio (binary outcome) or hazard ratio (time-to-event outcome) for level *j* compared to a reference level. We fit a linear regression model to the *r* ordered pairs with the median *x*_*j*_ of the covariate values within level *j* as the regressor and the effect size *θ*_*j*_ as the dependent variable. Trend tests between the ordinal covariate and the effect size may be conducted by testing whether the slope of the regression line is equal to zero against a two-sided alternative.

Typically, the procedure utilizes unweighted regression, which ignores variability in estimation of the effect size. Weighted least squares may be more appropriate and increase the efficiency of the estimator. One possible and commonly used weight [14] is the inverse of the estimated variance of the odds ratio or hazard ratio. Another popular method is to weigh each stratum estimate by the stratum sample size [15]. Additional methods are detailed in the Regression Methods section. In the following sections, we describe the simulation studies and data application. Sections detail components including the models, parameters, covariates, and outcomes. All simulations utilized SAS 9.2 [16].

### Sampling covariates

The simulated covariate was sampled from the 2007–2008 National Health and Nutrition Examination Survey (NHANES) [17]. Beginning in 1999, NHANES was given annually to a nationally representative sample of approximately 5000 non-institutionalized civilians per year. NHANES consists of interviews and physical examinations designed to assess the health of the U.S. population. A team of physicians and medical technicians conducted physical examinations in Mobile Examination Centers, standardized facilities that travel to survey locations throughout the country. Dietary and health interviewers asked additional questions using Computer-Assisted Personal Interview software.

Our exclusion criteria include pregnancy, missing BMI (*n* = 0), BMI above 70 (*n* = 4), and age less than 20 years (n = 0). The remaining 5551 eligible individuals had BMI values ranging from 14.2 to 67.3 kg/m^2^, a mean of 28.9 kg/m^2^ and standard deviation of 6.6 kg/m^2^. Table 1 includes summary statistics by weight class. The distribution of BMI is strongly right-skewed; less than 2% of the NHANES sample was classified as underweight. We oversampled the underweight class to ensure sufficient sample members for analysis. For each simulation, 80% of individuals in the sample were selected with replacement from the entire NHANES dataset, and the remaining 20% were selected from the subset consisting of underweight individuals.

### Binary outcome

We generate binary outcomes based on one of two models. The probability *p*_*i*_ that individual *i* with BMI *z*_*i*_ experiences the outcome is given by equation (1) or (2)

1 $$ \log \left(\frac{p_i}{1-{p}_i}\right)=\alpha +\beta \left({z}_i-25\right) $$

2 $$ \log \left(\frac{p_i}{1-{p}_i}\right)=\alpha +\beta {\left({z}_i-14.2\right)}^2 $$

Model (1) assumes a linear relationship between the logarithm of odds and BMI. The latter relationship is quadratic and monotone increasing for *z*_*i*_ > 14.2 kg/m^2^, the minimum value of BMI in the NHANES dataset.

Given α, β and *z*_*i*_, we calculate the expected event probability *p*_*i*_. We generated a random variate from the uniform (0,1) distribution, and let *y*_*i*_ = 1 if *u*_*i*_ *< p*_*i*_*, y*_*i*_ = 0 otherwise. Then we created ordered categories corresponding to the weight classes defined in [13] and fit model (3) to the outcome based on the weight class, using the “normal weight” class as the reference group.

3 $$ \log \left(\frac{p_i}{1-{p}_i}\right)={\beta}_0+{\beta}_1{x}_{\mathrm{i}1}+{\beta}_2{x}_{i2}+{\beta}_3{x}_{\mathrm{i}3} $$

Here, *x*_*ij*_ indicates whether individual *i* is in the *j*th ordered weight class. Underweight, overweight, and obese correspond to weight classes *j* = 1, 2, 3, respectively. We recorded the estimated odds ratios, *θ*_*j*_ *= OR*_*j*_ = exp.(*β*_*j*_) and the variances of the log-effect size, σ_j_^2^ = Var (*β*_j_) for j = 1, 2, 3.

We regressed the odds ratio, *θ*_*j*_, centered at 1, on the median, *m*_*j*_, centered at 23, of the BMI values within covariate class *j*. This invokes the linear model,

4 $$ {\theta_j}^{\ast }={\gamma}_0+{\gamma}_1{m^{\ast}}_j+{\varepsilon}_j $$

where *ε*_*j*_ is normally distributed*, E*(ε_j_) = 0, *θ*_j_^*^ *= θ*_*j*_ *− 1*, and m^*^_j_ *= m*_*j*_ – 23. The variance of *ε*_*j*_ is specified for each method in the “Regression Methods” section. We also investigated models without an intercept, i.e.

5 $$ {\theta_j}^{\ast }={\gamma}_1{m^{\ast}}_j+{\varepsilon}_j $$

Intuitively desirable, these models force the expected odds ratio for the referent group to be unity. For each method, we record whether the trend test for H_0_: *γ*_1_ = 0 was rejected at the 0.05 level.

### Time-to-event outcome

Given the log-hazards ratio *β* and the covariate *z*_*i*_ for individual *i*, we assumed one of two underlying proportional hazards models [18]. The hazard for individual *i* at time *t* can be modeled by either

6 $$ \Lambda \left({t}_i|{z}_i\right)={\Lambda}_0\left({t}_i\right)\exp \left[\beta \left({z}_i-25\right)\right] $$

or

7 $$ \Lambda \left({t}_i|{z}_i\right)={\Lambda}_0\left({t}_i\right)\exp \left[\beta {\left({z}_i-14.2\right)}^2\right], $$

assuming either a linear or a monotone quadratic relationship between the hazard rate and BMI. We generated event times *T*_*i*_ from an exponential distribution with hazard rates exp.[*β*(*z*_*i*_ – 25)] and exp.[*β*(*z*_*i*_ – 14.2)^2^] respectively [19]. Censoring times *C*_*i*_ were generated from the uniform (0, *b*_*k*_) distribution, where *b*_*k*_ is chosen so that the event probability for the entire sample is approximately *p*_*k*_ for *k* = 1, 2, 3. These probabilities correspond to heavy censoring (*p*_*1*_ = 0.2), medium censoring (*p*_*2*_ = 0.5), and light censoring (*p*_*3*_ = 0.7). The values of *b*_*k*_ are given in Table 2. The observed data for each individual include *v*_*i*_ = min (*t*_*i*_*, c*_*i*_) and *δ*_*i*_ = *I*(*t*_*i*_ < *c*_*i*_). We fit a Cox proportional hazards model [18] with the normal weight group as the reference group:

8 $$ \Lambda \left({t}_i|{x}_{i 1},{x}_{i 2},{x}_{i 3}\right)={\Lambda}_0\left({t}_{ij}\right)\exp \left({\beta}_1{x}_{\mathrm{i}1}+{\beta}_2{x}_{\mathrm{i}2}+{\beta}_3{x}_{\mathrm{i}3}\right) $$

**Table 2:** Values of b_k_ for the Uniform Censoring Intervals (0,b_k_)

Type 1 Error and Linear Alternatives					
k	p_k_	β = 0	β = log (1.01)	β = log (1.05)	β = log (1.1)
1	0.2	0.475	0.4	0.375	0.32
2	0.5	1.755	1.7	1.55	1.4
3	0.7	3.450	3.5	3.2	3.2
Quadratic Alternatives					
k	p_k_		β = 0.001	β = 0.002	β = 0.003
1	0.2		0.35	0.265	0.205
2	0.5		1.35	1.12	0.95
3	0.7		2.8	2.35	2.05

We record the resulting hazard ratios, *θ*_*j*_ *= HR*_*j*_ = exp.(*β*_j_) and the variances of their logarithms σ^2^_j_ = Var (*β*_j_). Then, we fit linear models given by equations (4) and (5) and recorded whether or not the trend test for H_0_: *γ*_1_ = 0 was rejected against a two-sided alternative.

### Parameter values

To quantify the type I error, we generated binary outcomes with *β* = 0, which reduces to.

$\log \left(\frac{p_i}{1-{p}_i}\right)=\alpha$, or *p*_*i*_ = exp.(α)/[1 + exp.(α)] . We investigated α = log (0.2), log (0.3), log (0.4), log (0.5), log (0.6), log (0.7), log (0.8). Each configuration was simulated 10,000 times with 1000 observations.

We computed power under the alternative that the effect size is linearly related to the covariate, i.e. we generated data according to equation (1) and (6) and β = log (1.01), log (1.05), and log (1.1). For an increase of 5 units in body mass index, these correspond to hazards ratios or odds ratios of about 1.05, 1.28, and 1.61, respectively.

We generated data according to equations (6) and (7) with the same β values detailed in the previous paragraph. The covariate is centered at 14.2, the minimum BMI value in the NHANES dataset, in equations (2) and (7). We chose to generate data in this form because it represents a monotone nonlinear increasing relationship with body mass index. All power estimates utilized 30,000 simulations, each of which used a sample size of 1000.

### Regression methods

We examine ten regression methods, seven weighted and three unweighted. We used the delta method for methods 4 and 5 to find the estimated variance of each effect size *θ*_*j*_, *j* = 1, 2, 3. Models lacking an intercept force the regression line to pass through the point with median 23 and effect size 1; the odds ratio or hazards ratio for the reference group is exactly 1. Models with an intercept lack this restriction. Models either used observations for all covariate levels or excluded the point corresponding to the reference group. Details are found in Table 3.

**Table 3:** Summary of Methods and Results: Type I Error and Power

Method	1	2	3	4	5	6	7	8	9	10
Weighted?	No	No	No	Yes	Yes	Yes	Yes	Yes	Yes	Yes
Weights	N/A	N/A	N/A	(*θ*_*j*_^*2*^*σ*^*2*^_*j*_)^*−1*^	(*θ*_*j*_*σ*_*j*_)^*−1*^	(*σ*^*2*^_*j*_)^*− 1*^	(*σ*_*j*_)^*− 1*^	*n*_*j*_	*n*_*j*_	*n*_*j*_
Intercept?	No	Yes	Yes	Yes	Yes	Yes	Yes	No	Yes	Yes
Reference Group included?	Yes	Yes	No	Yes	Yes	Yes	Yes	Yes	No	Yes
Type I Error Controlled?	No	Yes	Yes	Yes	Yes	Yes	Yes	No	Yes	Yes
Power: Linear	N/A	M	L	VL	VL	H	M	N/A	H	M
Power: Weak Quadratic	N/A	H	M	L	L	M	M	N/A	M	H
Power: Strong Quadratic	N/A	M	L	VL	VL	H	M	N/A	H	H

*VL* Very Low Power, *L* Low Power, *M* Moderate Power, *H* High Power

Relative amounts of power refer to the relative ranks of the methods. Methods classified as with high power had the most power for at least one scenario in the corresponding type of simulation. Methods classified as with medium or low power were ranked next. Methods classified with very low power displayed the lowest power in every simulation of that type

### Application of methods to NHANES data

We conducted trend tests between BMI and two outcomes in the NHANES dataset. The outcomes were based on the questions “Has your doctor told you that you have diabetes?” and “Has your doctor ever told you that you had high blood pressure?” Answer choices were “yes”, “no”, or “don’t know”. Responses of “don’t know” were considered missing. The diabetes question included the choice “borderline or pre-diabetes”, which we grouped with the “yes” category.

## Results

First, we report which methods are valid. Second, we investigate power of the valid methods based on various effect sizes. Finally, we provide a summary table comparing the methods (Table 3).

### Type I error

**Fig. 1:**
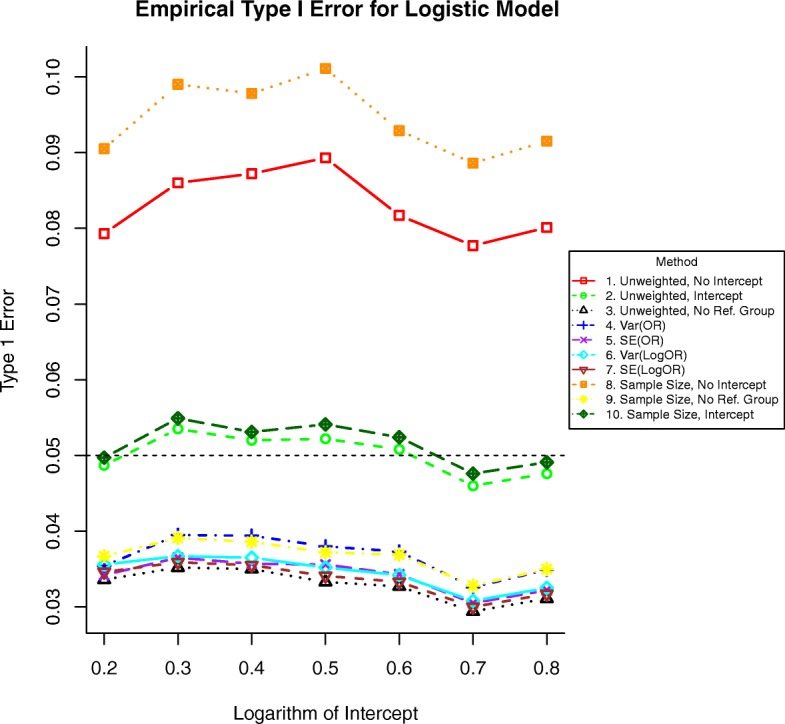
Simulated type I error rates for each method with binary outcomes by increasing intercept in Eq. (1), which in this case is equal to the log-odds of the event for a person with a BMI of 25 kg/m^2^.The dashed horizontal line at 0.05 indicates the significance level

**Fig. 2:**
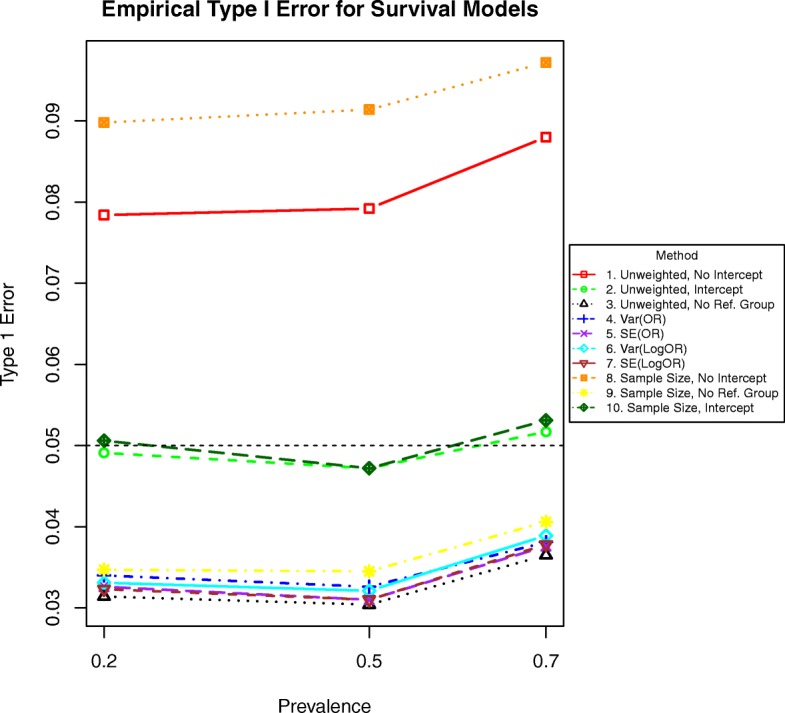
Simulated type I error rates for each method by increasing prevalence with time-to-event outcomes, or equivalently, decreasing censoring. The dashed horizontal line at 0.05 indicates the significance level

Both methods (1 and 8) without intercepts failed to control type I error. Figures 1 and 2 show consistently inflated size for these methods, which should not be used in practice.

The remaining unweighted regression methods (2 and 3) were valid, as were methods (6 and 7) weighted by the inverse variance or standard error of the logarithm of the effect size and the intercept-included methods that weight by the stratum sample size.

Simple unweighted regression (method 2) and simple regression weighting by the stratum sample size (method 10) had type I error near the nominal rate. All other methods were excessively conservative.

### Power

Before describing the results of the power analyses, we provide the reader with intuition. When the relationship between the effect size and ordinal covariate is weak, we expect that power should be low for all methods. For such weak relationships, we expect that proportion of events in each class would not be that different and the variances of the effect sizes should be close to each other. Consequently, homogeneity of variance is almost satisfied, and unweighted methods may be comparable to weighted methods.

**Fig. 3:**
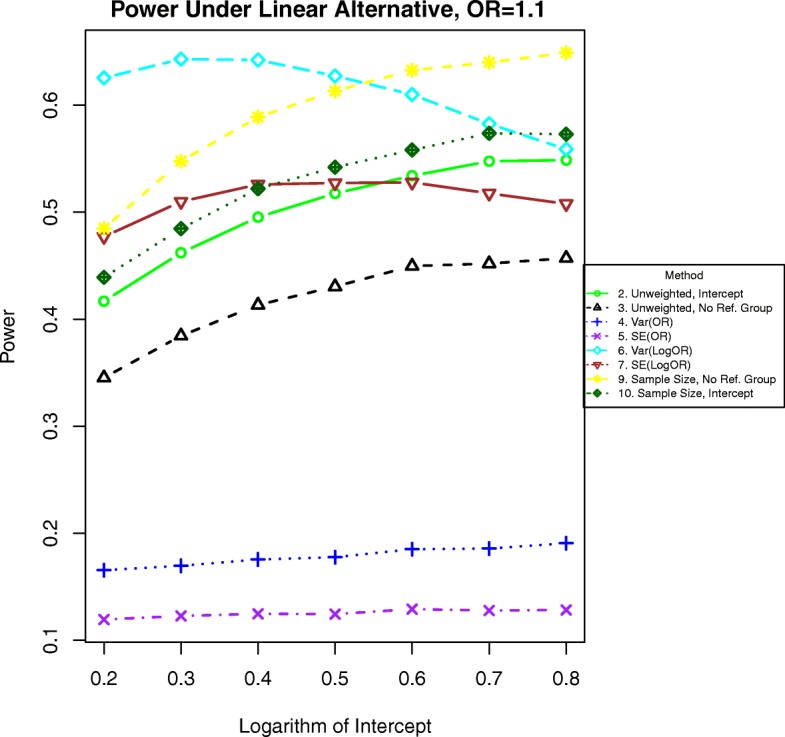
Power for binary outcomes generated by Eq. (1) with a linear alternative of an odds ratio of 1.1 for a unit increase in BMI

**Fig. 4:**
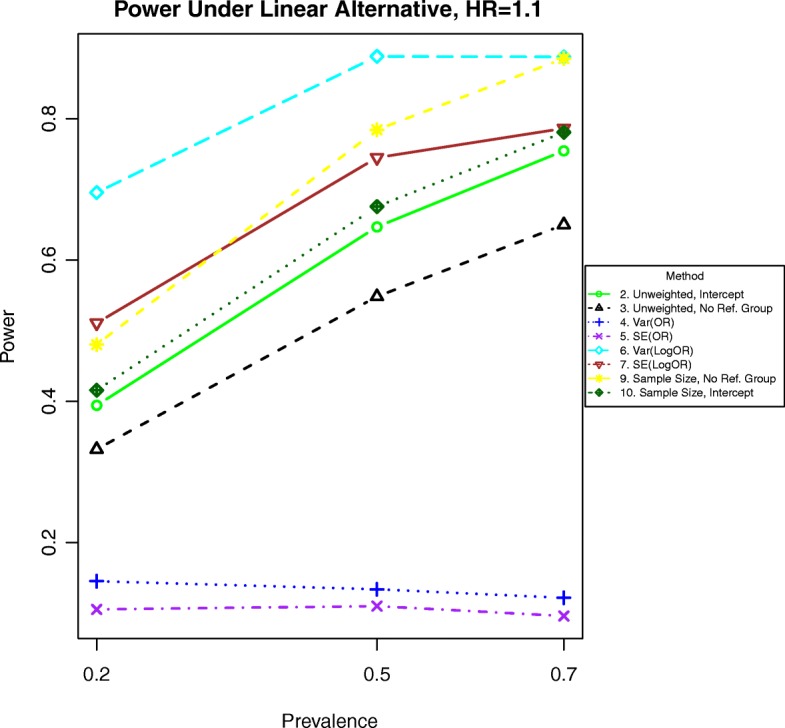
Power for time-to event outcomes generated by Eq. (6) with a hazard ratio of 1.1 for a unit increase in BMI

**Fig. 5:**
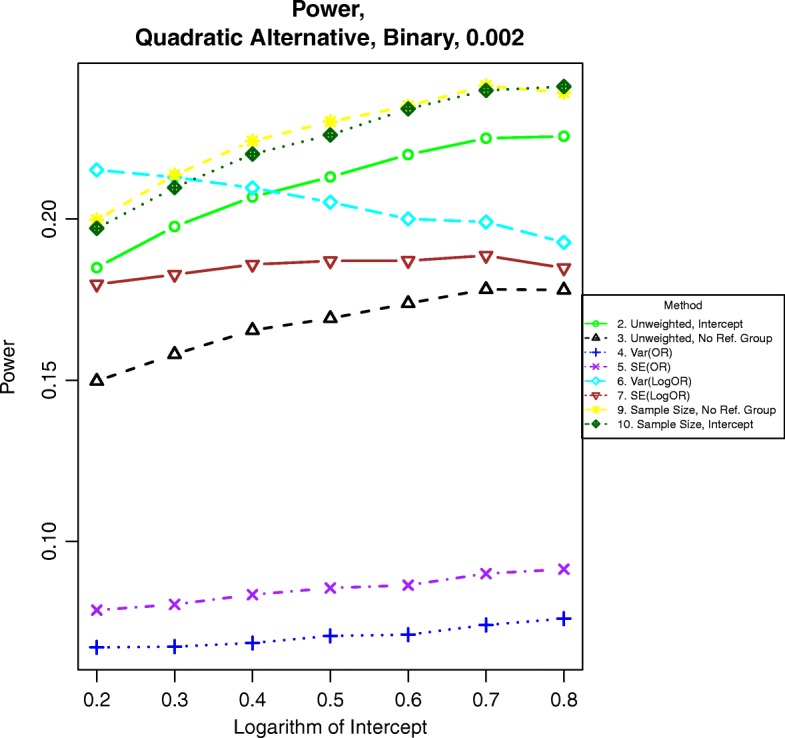
Power for binary outcomes generated by Eq. (2) with β = 0.001. The setup denotes a weaker quadratic relationship between the log-odds of the event and increasing BMI

**Fig. 6:**
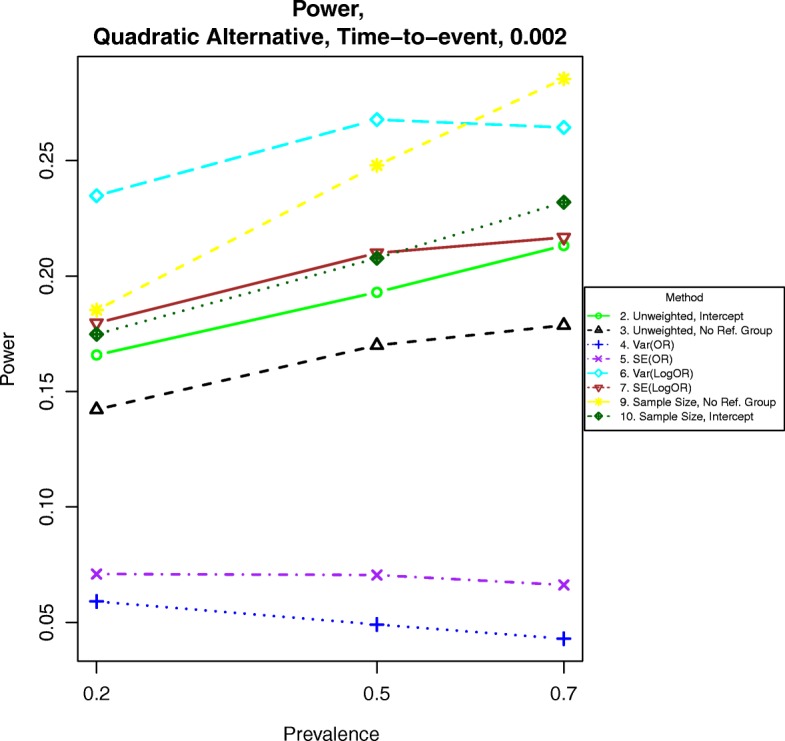
Power for time-to-event outcomes generated by Eq. (7) with β = 0.001. The setup denotes a weak quadratic relationship between the log-hazard ratio and BMI

**Fig. 7:**
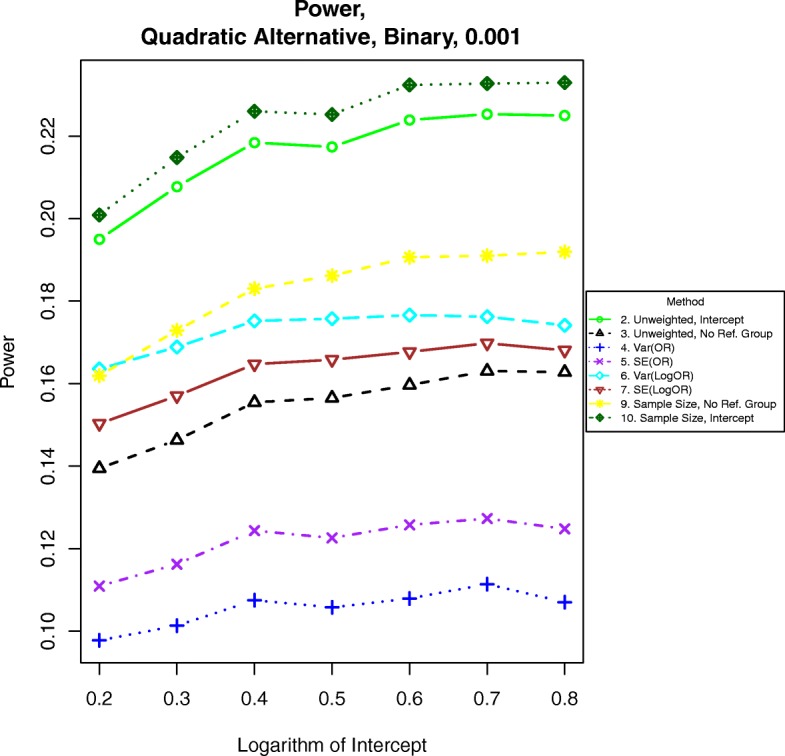
Power for binary outcomes generated by Eq. (2) with β = 0.002. The setup denotes a moderate quadratic relationship between the log-odds of the event and BMI

**Fig. 8:**
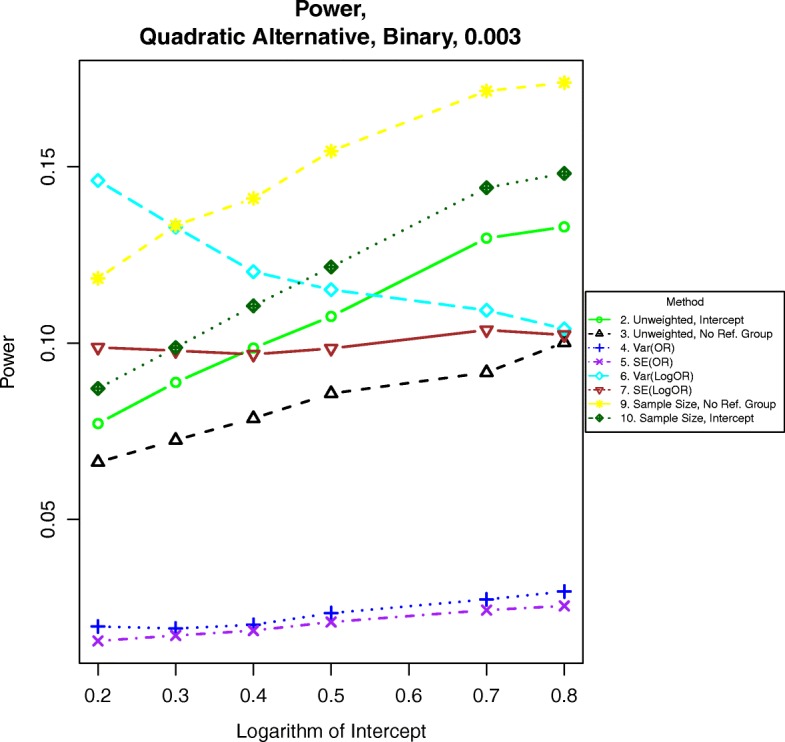
Power for time-to-event outcomes generated by Eq. (7) with β = 0.002. The setup denotes a strong quadratic relationship between the log-hazard ratio and BMI

**Fig. 9:**
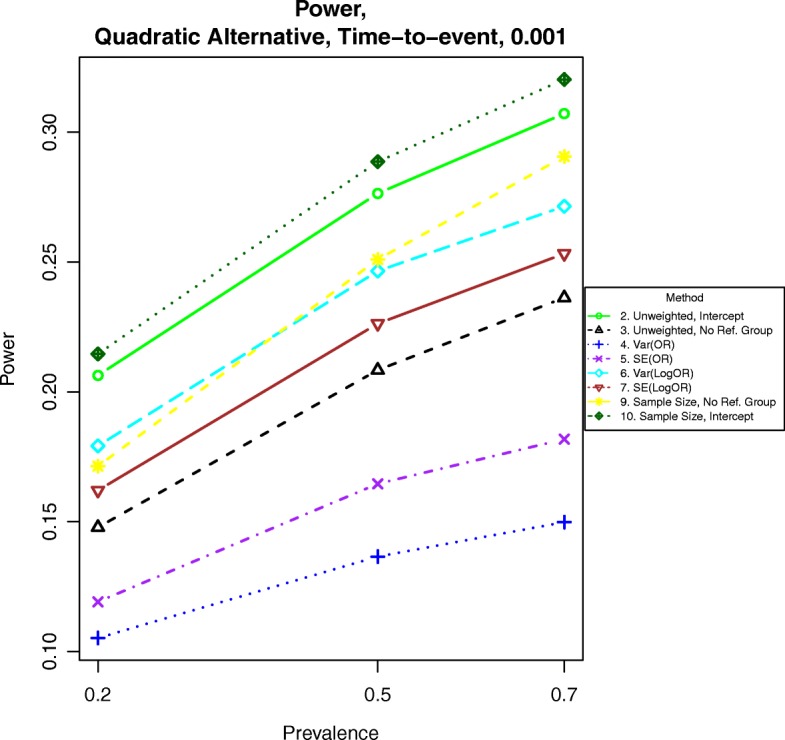
Power for binary outcomes generated by Eq. (2) with β = 0.003. The setup denotes a strong quadratic alternative, meaning the log-odds of the event increases quadratically with increasing BMI

**Fig. 10:**
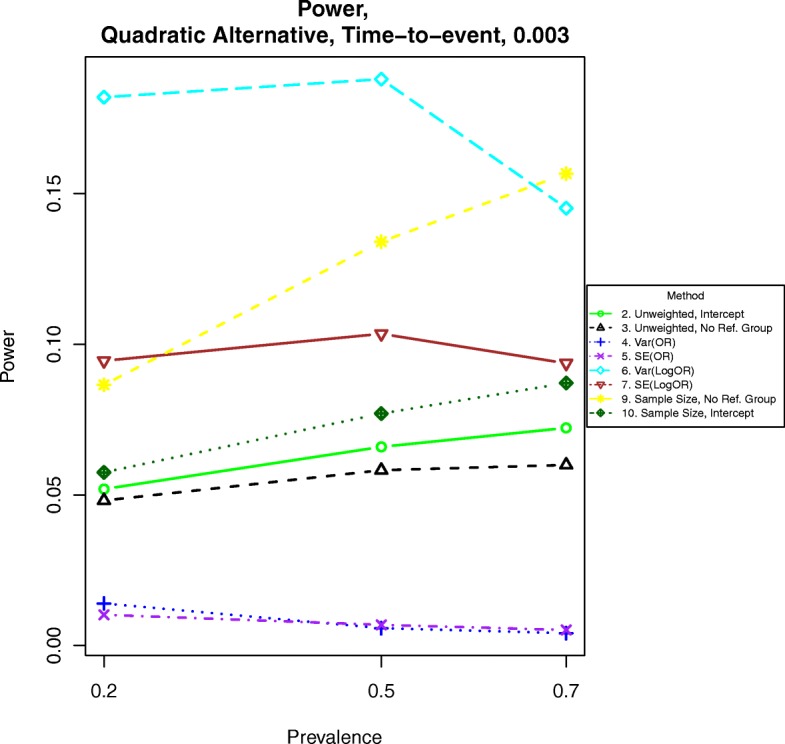
Power for time-to-event outcomes generated by Eq. (7) with β = 0.003. The setup denotes a strong quadratic alternative in which the logarithm of the hazard ratio increases quadratically with increasing BMI

By contrast, when the relationship between the effect size and the covariate is strong, we expect to observe increased power. The odds ratios should increase more quickly, and the variances may differ appreciably from each other. Therefore, we expect weighted methods to be more powerful than unweighted methods, as shown in Figs. 3 and 4. Power for quadratic alternatives are detailed in a subsequent section and Figs. 5, 6, 7, 8, 9, and 10.

### Power under linear alternative

For binary outcomes when the odds ratio is 1.1, the best methods are 6, which weights by the inverse of the variance of the parameter estimate and 9, which has excludes the referent group and weights by the stratum sample size. See Fig. 3. Simple unweighted regression (method 2) has similar power as the intercept method weighted by the stratum sample size (method 10), the unweighted method excluding the reference group (method 3) has slightly less power, and all other methods are less powerful.

Results are similar but not identical for time-to-event data. For hazards ratios 1.1 or greater, method 6 is the most powerful, as shown in Fig. 4. Under light censoring and a hazards ratio of 1.1, method 9 has about the same power. When the hazards ratio is 1.05, the preceding methods are still most powerful, but the methods weighting by the inverse of the variance of the logarithm of the hazards ratio (method 6) and the stratum sample size for the non-reference groups (method 9) are nearly as powerful.

**Fig. 11:**
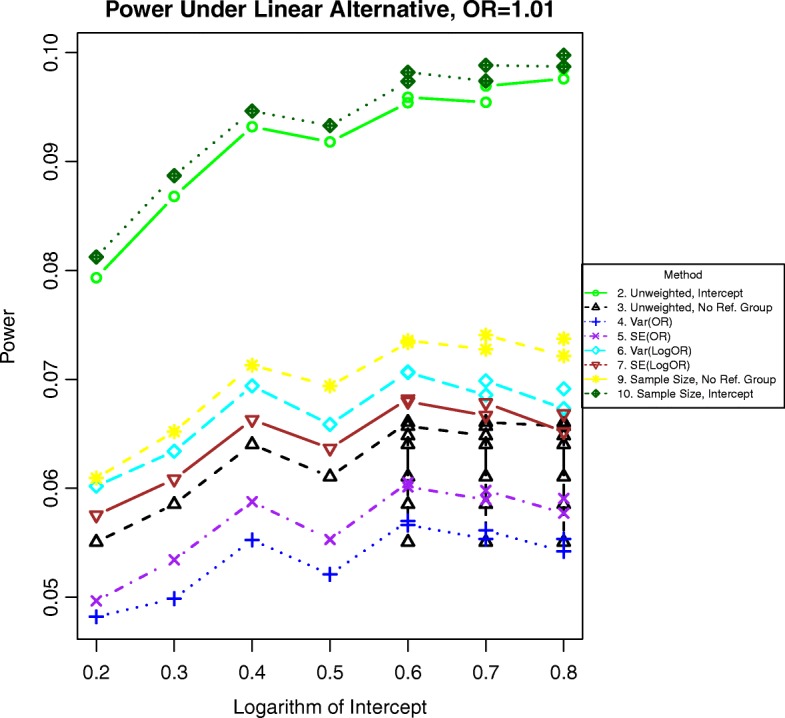
Power for binary outcomes generated by Eq. (1) with a linear alternative of an odds ratio of 1.01 for a unit increase in BMI. The setup denotes a weak linear alternative in which the log-odds of the event increases linearly but slowly with increasing BMI

**Fig. 12:**
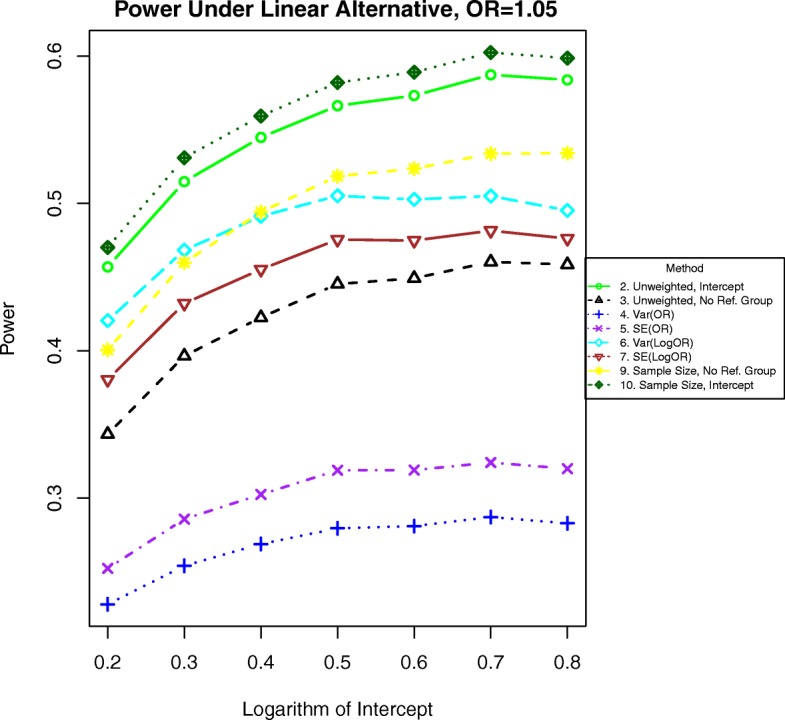
Power for binary outcomes generated by Eq. (1) with a linear alternative of an odds ratio of 1.05 for a unit increase in BMI. The setup denotes a weak linear alternative in which the log-odds of the event increases linearly at a moderate rate with increasing BMI

**Fig. 13:**
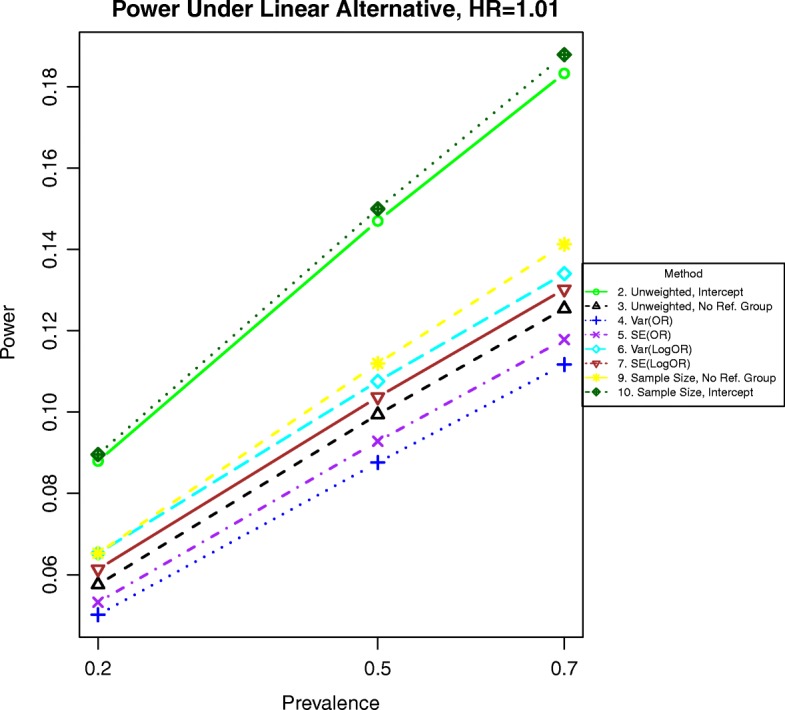
Power for time-to event outcomes generated by Eq. (6) with a hazard ratio of 1.01 for a unit increase in BMI. The setup denotes a weak linear alternative in which the logarithm of the hazard ratio increases linearly but slowly with increasing BMI

**Fig. 14:**
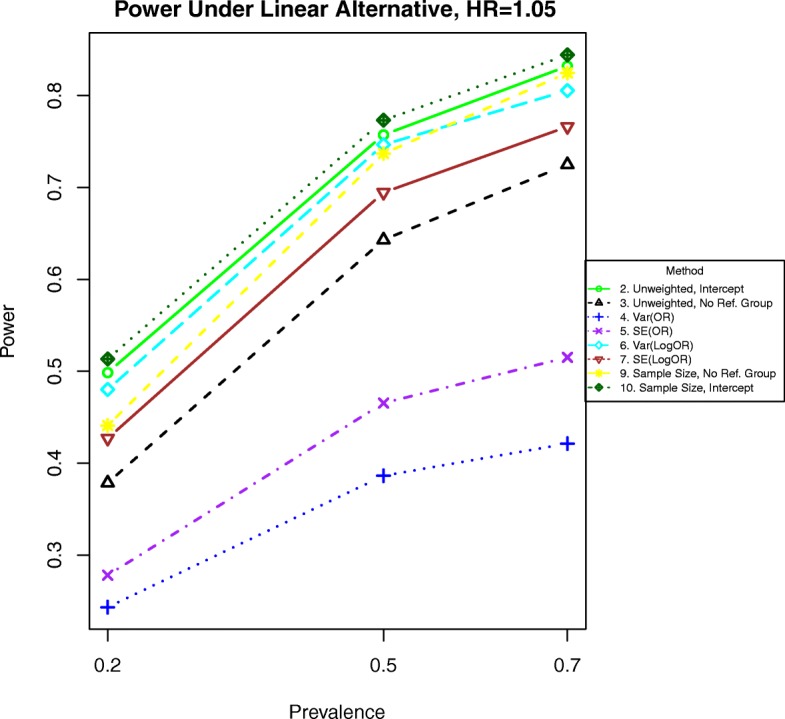
Power for time-to event outcomes generated by Eq. (6) with a hazard ratio of 1.05 for a unit increase in BMI. The setup denotes a weak linear alternative in which the logarithm of the hazard ratio increases linearly at a moderate rate with increasing BMI

Additional simulations for odds ratios of 1.01 and 1.05 are included in Figs. 11, 12, 13, and 14. In these simulations, the power is lower overall for all methods. For both binary and time-to-event outcomes, the power was highest for the method weighting by the stratum sample size (10) and the standard unweighted method (2), followed by the other weighted methods (6, 7, and 9). The improved ranking for the unweighted method 2 is likely due to the lack of events in each stratum and more homogeneous variances, which decrease the need for complex weighting in these cases.

### Power under monotone quadratic alternative

For power calculations under quadratic alternatives as shown in eqs. (2) and (7), we examined β = 0.001, 0.002 and 0.003. These correspond to odds ratios or hazards ratios of about 1.12, 1.25 and 1.40, respectively for a BMI of 28 compared to a BMI of 23. Power under nonlinear alternatives is shown in Figs. 5, 6, 7, 8, 9, and 10. In contrast with the linear alternative, unweighted regression was nearly always less powerful than the best weighted method. When the effect size is related to body mass index through a weak quadratic relationship, such as β = 0.001, either method 3, 6, 9, or 10 is the most powerful method, depending on the intercept for binary data or the prevalence for time-to-event data. This observation holds for all cases for binary data and all three levels of censoring for time-to-event data. When β = 0.002 or β = 0.003, method 9 is the most powerful in most cases for binary data and under time-to-event data with light censoring. Method 6 is the most powerful for binary data when β = log(0.2) and time-to-event data under heavy or medium censoring when β = 0.002 or β = 0.003. The different ranking of each method is likely explained by the number of events present in the dataset in each group. This is evident by the fact that results are similar between binary data with β = log(0.2), corresponding to a prevalence of about 17% for those with a BMI of 25, and heavy censoring, corresponding to an event probability of 0.2.

### Application to NHANES data

Weighted frequencies for the outcomes are included in Table 4. None of the underweight individuals have diabetes, and only one underweight individual (0.33% weighted) has borderline or pre-diabetes. High blood pressure has sufficient numbers for all weight classes. Results are summarized in Table 5. All tests were rejected for diabetes, meaning that the prevalence of diabetes increases with increasing BMI class. The test for high blood pressure was not rejected for methods 2, 3, 4 or 5. This is not surprising; methods 4 and 5 were the least powerful in all simulations. While these trend tests were not significant, the *p*-values were just above 0.05. By contrast, tests for trend for high blood pressure were rejected by the remaining weighted methods, meaning that the prevalence of high blood pressure increases with increasing BMI class. The relative magnitude of the p-values in these tests parallel those in the simulations; results are similar between the methods that weight by the inverse variance or standard error of the log-odds ratio, or by the stratum sample size, with or without using the normal weight group.

**Table 4:** Cell Counts (and Weighted^a^ Percentage) of Outcomes within Each Weight Class in the NHANES Dataset

Outcome	Underweight	Normal Weight	Overweight	Obese	All
Diabetes Status					
No Diabetes	92 (99.7)	1406 (95.3)	1699 (93.0)	1563 (81.8)	4760 (90.0)
Diabetes or Pre/Borderline	1 (0.3)	105 (4.7)	219 (7.0)	460 (18.2)	785 (10.0)
Blood Pressure Status					
No High Blood Pressure	74 (83.5)	1153 (81.1)	1275 (70.4)	1050 (57.1)	3552 (69.4)
High Blood Pressure	19 (16.5)	359 (18.9)	642 (29.6)	974 (42.9)	1994 (30.6)

^a^Weighted percentages take into account the sampling design used in NHANES

**Table 5:** Conclusions of Trend Tests for the NHANES Dataset

Outcome	Method	1	2	3	4	5	6	7	8	9	10
Diabetes or Pre/Border-line	P-value	N/A	0.024	0.023	0.032	0.034	0.010	0.013	N/A	0.012	0.020
	Reject	N/A	Yes	Yes	Yes	Yes	Yes	Yes	N/A	Yes	Yes
High Blood Pressure	P-value	N/A	0.051	0.064	0.056	0.061	0.010	0.023	N/A	0.007	0.016
	Reject	N/A	No	No	No	No	Yes	Yes	N/A	Yes	Yes

Yes = The null hypothesis of no trend was rejected at the 0.05 significance level

No = The null hypothesis of no trend was not rejected at the 0.05 significance level

Method 1: Unweighted, no intercept

Method 2: Unweighted, with intercept

Method 3: Unweighted, with intercept, reference group omitted

Method 4: Weighted by the inverse of Var (OR), with intercept

Method 5: Weighted by the inverse of SE (OR), with intercept

Method 6: Weighted by the inverse of Var (LogOR), with intercept

Method 7: Weighted by the inverse of SE (LogOR), with intercept

Method 8: Weighted by the sample size, no intercept

Method 9: Weighted by the sample size, with intercept, reference group omitted

Method 10: Weighted by the sample size, with intercept

## Discussion

Regression methods without intercepts had inflated type I error. The relative power of the remaining eight methods varies with the underlying relationship and the number of events in each group. To implement these methods, standard sample size requirements must be met. There should be at least 10 subjects in each stratum experiencing the outcome of interest and 10 subjects in each stratum not experiencing the outcome [6]. Insufficient sample size may result in inflated probability of type I error or loss of power.

The odds ratio variance estimate depends on both the variance of the logistic regression slope parameter and the square of the odds ratio itself. Consequently, in these simulations, when holding the parameters from logistic regression constant and assuming that prevalence is increasing with increasing covariate value, the odds ratio variance estimate for higher BMI classes exceeds the corresponding variance for the lower BMI classes. This relationship is more pronounced as the effect size increases and the ratio estimates become more extreme. Dependence of the variance of the ratio estimates on the two aforementioned sources of variability results in lower power once the true effect size exceeds about 1.1 based on the results of these simulations. Naturally, the methods that depend only on the variance of the parameters from logistic regression have much higher power. In fact, method 6 was one of the methods with the highest power in most cases when the effect size was moderate or large. Similarly, weighting by the stratum sample size results in adequate power because the variances are approximately proportional to the stratum sample sizes. In these cases, unweighted methods perform worse because they fail to account for heterogeneity in the variance of the estimates in each stratum. Unweighted methods, on the other hand, performed well when the effect size was small and the variances were more similar.

Applying the methods to NHANES showed a trend of increasing diabetes with increasing BMI. For high blood pressure, four valid weighted trend tests were significant, while the two valid unweighted trend tests failed to achieve significance. Fitting a logistic regression model to the NHANES dataset for each outcome indicates that the outcomes are at least moderately linearly associated with the median of the BMI class. (Adding quadratic terms did not significantly improve the model based on a type III test.). Simulations indicate that most powerful and type-I error controlled methods for binary outcomes with linear alternatives are methods 6 and 9, depending on the intercept of the model. Based on the weighted prevalence estimates for diabetes and high blood pressure, the intercept should be around 0.2 or less, indicating that method 6 is preferred. Indeed, for both outcomes, method 6 rejected the trend test, indicating that the diseases are associated with increasing BMI class.

While it is conceptually reasonable to force the effect size for the reference group to be unity, methods with this restriction were not valid. On the other hand, the methods excluding the reference group seems counterintuitive. Instead of having 4 data points, only the 3 non-referent points are included in the model. Yet, these method were powerful when the effect sizes displayed certain nonlinear trends.

The study includes a variety of simulations, but it is subject to limitations. We considered various situations that are common in practice in our simulations, but there could be other situations that worth exploring in the future. The situations that we considered assumed observations to be independent. Correlated data could arise in practice and it will be of interest to examine the performance of the various tests with correlated observations in future research.

In conclusion, under most situations, trend tests between an ordinal covariate and an odds ratio or hazard ratio should employ either simple unweighted regression or regression weighted by either the stratum sample size (for a small effect size) or the inverse variance of the logarithm of the effect size (for a large effect size). If there is reason to believe that the relationship between the ordinal covariate and the effect size increases more quickly than a linear relationship, it would be best to fit the data to a regression model without the reference group and weighted by either the stratum sample size or the inverse variance of the logarithm of the effect size.

## Conclusions

The hypothesized relationship between the discretized covariate and the outcome should be considered carefully to ensure that trend tests have adequate type I error and power. Trend tests based on regression models forced to pass through the point with the null value at the median for the reference group are intuitive but not statistically valid. For most cases, we recommend using a trend test based on either simple unweighted regression between the discretized covariate and the outcome, or regression weighted by either the stratum sample size or the variance of the logarithm of the effect size. The importance of the study is its ability to serve as a guide for future analysts throughout medicine, epidemiology, and public health to select an appropriate trend test for binary or time-to-event outcomes and ordinal covariates.
